# The clinical characteristic of catathrenia: a new look at an old issue—a systematic review of existing literature

**DOI:** 10.1007/s11325-024-03033-0

**Published:** 2024-05-17

**Authors:** Bartlomiej Blaszczyk, Adam Wichniak, Mieszko Wieckiewicz, Anna Brzecka, Dorian Nowacki, Monika Michalek-Zrabkowska, Gabriella Lachowicz, Grzegorz Mazur, Helena Martynowicz

**Affiliations:** 1 Student Research Club No K133, Faculty of Medicine, Wroclaw Medical University, 50-556, Wroclaw, Poland; 2 Third Department of Psychiatry and Sleep Medicine Centre, Institute of Psychiatry and Neurology, 02-957 Warsaw, Poland; 3 Department of Experimental Dentistry, Wroclaw Medical University, 50-425, Wroclaw, Poland; 4 Department of Pulmonology and Lung Cancer, Wroclaw Medical University, 53-439, Wroclaw, Poland; 5 Department of Human Nutrition, Wroclaw University of Environmental and Life Sciences, 51-630 Wroclaw, Poland; 6 Department of Internal Medicine, Occupational Diseases, Hypertension and Clinical Oncology, Wroclaw Medical University, 50-556 Wroclaw, Poland

**Keywords:** Sleep stages, Nocturnal groaning, Nocturnal moaning, Sleep disorders, REM cycle, NREM cycle

## Abstract

**Study objectives:**

The International Classification of Sleep Disorders categorized catathrenia as a respiratory disorder, but there are doubts whether episodes appear during rapid eye movement (REM) sleep or the non-rapid eye movement (NREM), their duration, and symptoms. The main objectives were to identify the most common features and relations of catathrenia.

**Methods:**

PubMed, Embase, and Web of Science were searched according to the PRISMA 2020 guidelines. The Joanna Briggs Institute and the ROBINS-I tools were chosen to assess the risk of bias.

**Results:**

A total of 288 records were identified, 31 articles were included. The majority of the studies had a moderate risk of bias. 49.57% of episodes occurred during the NREM sleep, while 46% took place during REM. In 60.34% females, catathrenia was more common in the NREM, while in 59.26% of males was in REM sleep (*p* < 0.05). Females and obese individuals were found to have shorter episodes (*p* < 0.05). Age was inversely correlated with minimal episodes duration (*r* =  − 0.34). The continuous positive airway pressure (CPAP) therapy was inversely correlated with the maximal episode duration (*r* =  − 0.48).

**Conclusions:**

Catathrenia occurs with similar frequency in both genders. The most frequent symptoms embraced groaning, awareness of disturbing bedpartners, and daytime somnolence—not confirmed by the Epworth Sleepiness Scale. The episodes occur more frequently in NREM than in REM sleep. Catathrenia may be considered as a sex-specific condition. The effects of CPAP treatment leading to shortening episodes duration, which may indicate the respiratory origin of catathrenia.

**Supplementary Information:**

The online version contains supplementary material available at 10.1007/s11325-024-03033-0.

## Introduction

Sleep disorders affect millions of people in the USA [1]. These problems can occur in up to 41% of young adult females and 42.3% of males [2]. Sleep disturbances are connected with serious diseases such as depression, dementia, strokes, migraine, and hypertension [3–7], and may subsequently increase the mortality rate among patients experiences these disorders [1, 8, 9]. Although insufficient awareness of sleep disorders exists in the general population [10], the symptoms of sleep problems, e.g., excessive daytime sleepiness, snoring, chronic fatigue, or impaired daytime functioning [11–14] have led to an increased number of doctor’s appointments. Subsequently there is observed a rise in the number of patients being diagnosed with sleep disorders [15]. However, atypical symptoms such as nocturnal groaning without awareness, as observed in catathrenia, could remain undiagnosed for many years [16]. Only when family or bed partners observe and complain of disturbing sounds during sleep, sleep evaluation will be finally conducted [17].

Catathrenia is characterized by the production of nocturnal groaning or moaning during expiration [18]. The sound of catathrenia is described as having rhythmic or semi-rhythmic formants that arise in the vocal cords. This is in contrast to a similar sound produced during snoring, which has instead been described as a sound with a guttural chaotic waveform that is produced both during inspiration and expiration [19, 20]. The differential diagnosis of nocturnal sounds includes sleep apneas, snoring, laryngospasm, stridor, and somniloquy (sleep talking) [21]. The global prevalence of catathrenia is unknown. However, it has been suggested that the problem involves approximately 0.063 to 0.54% of patients that have been referred to sleep specialists [19]. The name “catathrenia” itself was only established in 2001. The origin of the word “cathrenia” is Greek, with “threnia” meaning “to lament,” and “kata” meaning “below” [22]. One of the reasons for catathrenia being described so rarely in literature may be caused by closely resembles of sleep apnea and nocturnal moaning in polysomnography (PSG) recordings. These are difficult to differentiate, particularly when they are examined by inexperienced persons who do not have access to audio and video recordings [16]. Additionally, cases of catathrenia are also identified accidentally while screening for a sleep breathing disorder [23].

There is a significant amount of inconsistent data on the topic of catathrenia. First of all, catathrenia was initially classified by the International Classification of Sleep Disorders (ICD-2) as rapid eye movement (REM) related parasomnia. However, in the third edition of this classification (ISCD-3), it has been classified as a respiratory disorder [24]. In ISCD-3, catathrenia has been described as occurring predominantly during REM sleep. However, studies have emerged reporting episodes of catathrenia that occurred predominantly, or even only, during the NREM sleep [25–27]. Reported symptoms are also inconclusive, as some patients do not report any morning complaints [22, 27, 28], while some only report mild hoarseness [29]. Others however have reported symptoms that have severely affected their lives, such as excessive daytime sleepiness, unrefreshing sleep, choking episodes, and a decreased ability to concentrate during the day [16, 21]. The duration of the groaning sounds is in the range of 2–49 s according to ISCD-2 [30]. However, some studies in literature have described episodes as short as 0.4 s or as long as 154 s [25, 31, 32]. Several authors have reported the potential association between catathrenia and obstructive sleep apnea (OSA) [16, 33, 34]; however, still little is known about potential predisposing comorbid diseases for catathrenia [18].

This systematic review was performed to analyze the studies investigating catathrenia and to aggregate the existing data for a better understanding of this condition. The primary objective was to identify the most common features of catathrenia. The second objective was to determine the potential association between catathrenia and other diseases, gender, symptoms, or particular sleep stages.

## Methods

Our systematic review was conducted according to the criteria established by the Preferred Reporting Items for Systematic Reviews and Meta-Analyses 2020 (PRISMA 2020). This systematic review was not registered.

### Eligibility criteria

Inclusion criteria included single cases such as case reports and case series or cohort, cross-sectional, case–control, or descriptive studies where the detailed descriptions of patients experienced catathrenia were presented. This review took into consideration patients age, gender, symptoms, and the method in which this condition was diagnosed. No time constraint was placed on the publication date of the analyzed studies; however, the studies had to be written in English and were required to have full-text availability. Exclusion criteria were as follows: subjects unrelated to our topic; non-English articles; non-original records such as reviews, book chapters, letters to the editor and comments; papers that did not describe features of catathrenia; records containing the new catathrenia description, that could not be appropriately assessed for risk of bias; studies that could not be retrieved for evaluation.

### Information sources and searching strategy

In order to identify articles related to this systemic review, three medical databases were searched on May 5, 2023: PubMed, Embase and Web of Science. The following key terms were used to browse the databases: “catathrenia” OR “nocturnal groaning” OR “sleep-related groaning” OR “expiratory groaning” OR “nocturnal moaning” OR “nocturnal vocalization.” The initial search did not use any filters. Initially, the overall number of records were identified by two authors (BB and HM). Then, based on data obtained from the title and abstract, the duplicates, non-English studies, and papers unrelated to catathrenia were excluded. Next, full-text records were read by two authors (BB and HM) separately, who extracted the data and later compared the results. According to the eligibility criteria mentioned in the “Eligibility criteria” section, as well as the PRISMA 2020 guidelines, BB and HM excluded papers to reach studies that had focused on describing the catathrenia condition alone. If two authors (BB and HM) were in conflict during the inclusion process, the third author (MW) resolved this problem by means of a discussion.

### Data extraction

The following data was extracted from the included studies: the authors, type of study, number of patients, their gender, age, reported symptoms, their comorbidities, concomitant sleep disorders, points obtained in the Epworth Sleepiness Scale (ESS), methods used to diagnose catathrenia, the sleep stage in which the episode of moaning occurred, moaning duration, frequency of nocturnal episodes, groaning vocalization, and methods used to treat catathrenia. The extraction of the data was conducted independently by two authors (BB and HM).

### Risk of bias evaluation

Due to a large variety of included studies such as case reports, case series, cohort studies, and case–control studies, multi-adjustment tools had to be utilized. The Joanna Briggs Institute (JBI) critical appraisal tools were used for case reports and case series [35]. Non-randomized observational studies such as cohort and case–control studies however were analyzed with the ROBINS-I tool [36]. The possible answers to JBI questions were “yes,” “no,” “unclear,” and “not applicable.” The overall risk of bias could be low, moderate, or high. According to the JBI checklists, case reports were analyzed on the basis of 8 questions, while case series were analyzed using 10 questions. To be classified as having a low risk of bias, case reports had to receive at least 7 “yes” answers, while 9 “yes” responses were expected in the case series and cohort articles. For a study to be deemed as having a high risk of bias, a case report had to receive less than 5 “yes” answers, while case series papers had to receive less than 6 positive responses. Papers were considered to have a moderate risk of bias when answers were found to be between the abovementioned ranges. Using ROBINS-I, 7 domains of potential bias were determined. Judgements in each of the domains, as well as overall assessment, could be assigned as “low,” “moderate,” “serious,” or at “critical” risk of bias. A study was considered to have an overall low risk of bias in ROBINS-I if all domains were deemed to be at “low risk.” A “moderate risk” of bias was considered if a particular study had obtained either low or moderate risk responses in 7 domains. An overall “serious” or “critical risk” of bias was assigned when a particular study had received either of these categories in at least one domain. Evaluation for the risk of bias was assessed separately by two authors (BB and HM) who had compared the data.

### Statistical analysis

To reach the primary objective of our systematic review, extracted data was analyzed using Statistica 13.3 (Statsoft, Poland). Data is presented as a mean and standard deviation (SD), in some cases as weighted arithmetic mean and weighted SD. Data was analyzed using the Spearman rank correlation coefficient and chi-square *χ*^2^. The differences between two groups were analyzed with the non-parametric Mann–Whitney *U* test. For all analysis, *p* < 0.05 was considered to be statistically significant. Because of heterogenous characteristics being analyzed across the various studies, the number of variables (*n*) for each analysis can differ. The missing data was deleted pairwise and each result of the analysis is supported by the number of variables (*n*).

## Results

### Included studies

**Fig. 1:**
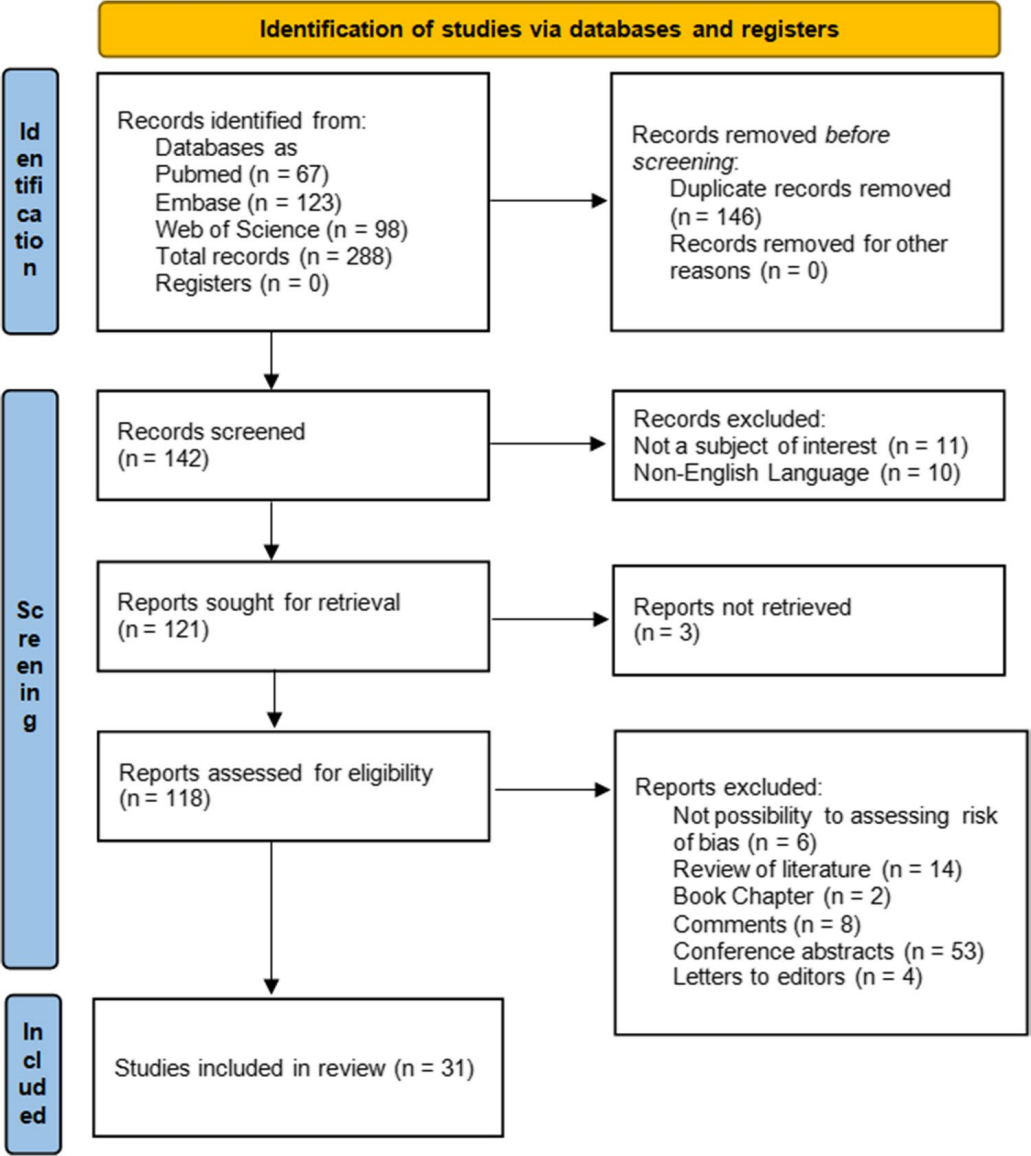
The PRISMA 2020 flowchart

After searching the key terms in three databases, we identified a total of 288 records: 67 in PubMed, 123 in Embase, and 98 in Web of Science. Next, 146 duplicates were removed. Following this 10 non-English papers, 11 papers unrelated to catathrenia and 3 records without full access were removed after the assessment of the title or abstract. Then, for the eligibility process, 118 full-text papers were read. According to the criteria laid out in Sect. "Eligibility criteria" and in accordance with PRISMA 2020 guidelines, 53 conference abstracts, 14 reviews, 4 letters to the editor, 2 book chapters, 8 comments, and 6 papers not fitting to any standard type of article, were excluded from the final analysis. Finally, 31 papers [16–19, 22, 25–28, 30–33, 37–54] had met the criteria of our study and were included in the review. The detailed steps of the study selection are presented in Fig. 1.

### Study characteristics

Thirty-one articles [16–19, 22, 25–28, 30–33, 37–54] were included for further analysis. These included 6 cohort studies [16, 26, 31, 42–44], 1 case–control study [39], 10 case series [19, 22, 24, 25, 37, 38, 40, 41, 45, 47], and 14 case reports [17, 18, 27, 28, 30, 32, 33, 46, 49–54]. However, in 3 studies [16, 25, 43], data was reported with SD and mean values. These were therefore excluded from further analysis. Without these 3 studies, the group of participants consisted of 127 patients, where there was a slight predominance of female (*n* = 69) over male patients (*n* = 58). The average age was 31.45 ± 14.66 years old. Unfortunately, as the studies were not homogenous in nature and different characteristics were evaluated across the papers, some studies did not contain the analyzed values that we mentioned; thus, our review always reports on the number of patients (*n*) who were included in the analysis of a particular feature. The overall body mass index (BMI) for all participants was 24.20 ± 5.73 kg/m^2^ (*n* = 76), with 13.16% of them being categorized as obese. These data are presented in Table 1. But all features of the included studies have been extensively shown in supplementary materials in Table 1.

**Table 1:** The overall characteristics of catathrenia patients

Feature	Mean	SD	Number of observations (*n*)
Male	58	––-	––-
Female	69	––-	––-
Male (%)	45.67%	––-	––-
Female (%)	54.33%	––-	––-
Total	127	––-	––-
Age	31.45	14.66	127
BMI	24.20	5.73	76
Obesity (%)	13.16%	––-	––-

*SD*, standard deviation; *BMI*, body mass index

### Clinical features of catathrenia

**Fig. 2:**
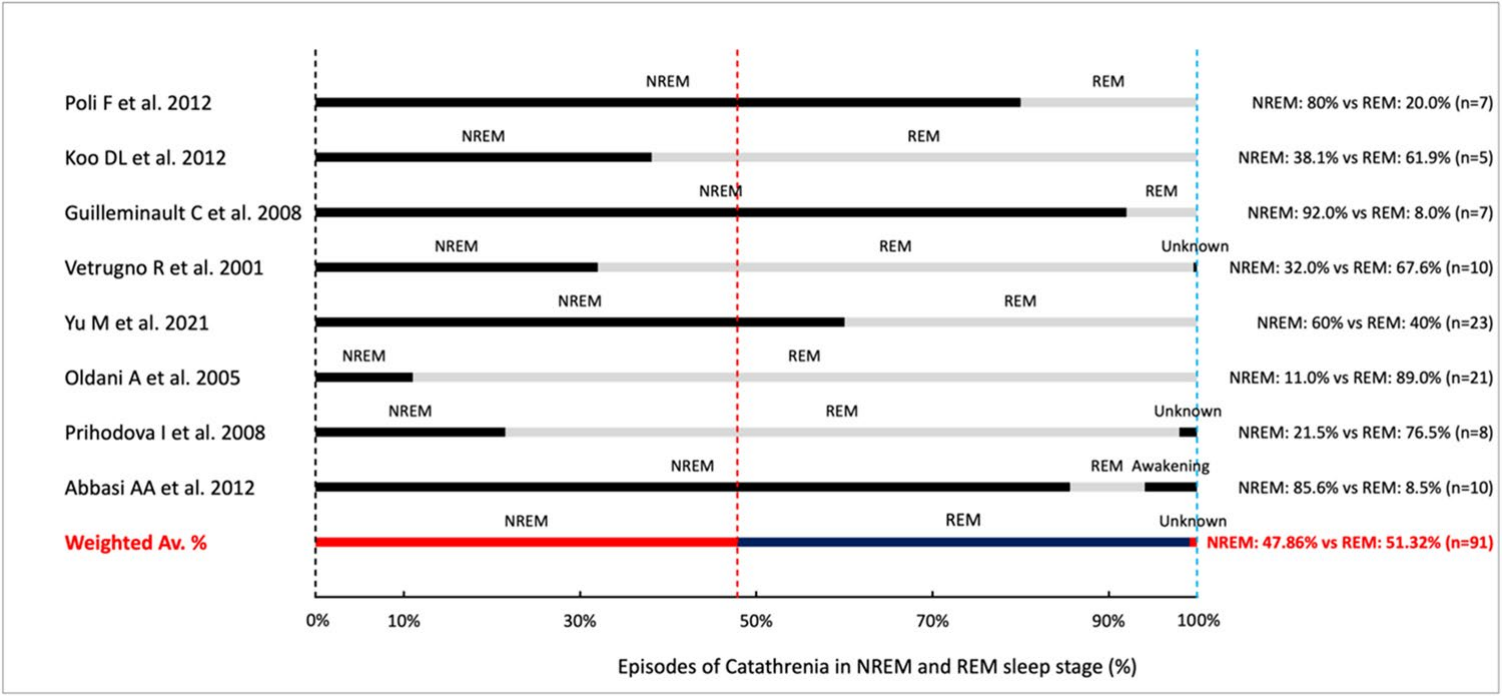
The graphic presentation of occurring catathrenia episodes in particular sleep phases

**Fig. 3:**
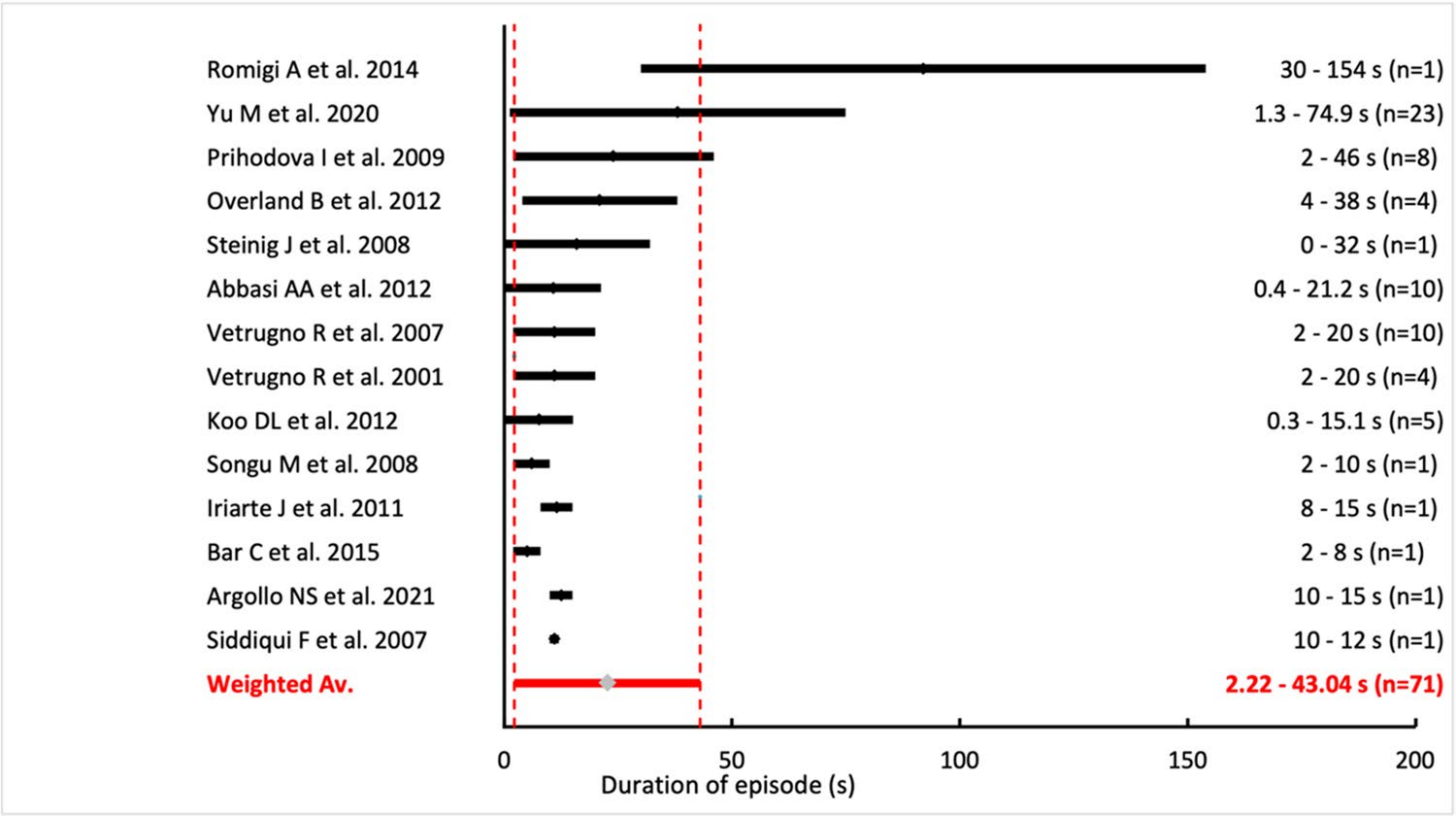
The graphic presentation of episodes duration in range minimum–maximum

Based on data obtained from the studies, we determined that catathrenia episodes occurred in 47.86% ± 30.46% in NREM sleep and 51.31% ± 31.16% in REM sleep (*n* = 91; Fig. 2). 6.10% of nocturnal groaning episodes were observed during awakenings from sleep (*n* = 10). In the assessment of the phase of sleep in which these episodes dominated, we found that in 49.57% cases episodes of catathrenia were more frequent in the NREM sleep, while 46% of cases they occurred more frequently in the REM cycle (*n* = 117). The minimal duration of episodes was determined to be 2.22 ± 3.81 s, while the maximal duration of the episodes was 43.04 ± 28.51 s (*n* = 71; Fig. 3). Forty-two out of 96 patients reported awareness about occurring episodes at their home, whereas 30 of them complained of episodes occurring every night. The precise vocalization of catathrenia was evaluated in only 50 patients. The most common type of analyzed vocalization during hospital examination was the type I sound with a sinusoidal waveform. The second was the type II sound with a semi-rhythmic sawtooth waveform.

The most frequently reported complaints included awareness of disturbing to bed partners, snoring, groaning, atypical nocturnal sounds, abnormal patterns of sleep breathing, and excessive daytime sleepiness (EDS) (*n* = 92). The EDS was estimated by the Epworth Sleepiness Scale (ESS). Mean ESS score was 5.78 ± 4.65 points (*n* = 107). Other frequently described complaints included nocturnal noises, family concerns about their health and the sensation of a dry mouth in the morning. Studies that focused on health concerns, determined that 46.58% of participants were healthy (*n* = 73). While focusing on concomitant sleep disorders, almost 20% of participants did not demonstrate any other sleep disorders. Of the remaining patients, 11.30% had insomnia, followed by less frequently occurring OSA and sleep bruxism (*n* = 115). It is worth noting though, that in almost 10% of the examined participants, other sleep diseases were not reported. To diagnose catathrenia, PSG was used in 122 patients. Abovementioned features of catathrenia are presented in Table 2. Treatment primarily involved CPAP therapy.

**Table 2:** The frequency of particular catathrenia features

Feature	Number of observations (*n*)	% of patients	Total number of patients (*n*)	Feature	Number of observations (*n*)	% of patients	Total number of patients (*n*)
Dominated sleep stage				Health status			
NREM	58	49.57%	117	Healthy	34	46.58%	73
REM	54	46.15%	117	Sleep disorders			
Not reported	10	7.87%	127	OSA	14	12.17%	115
Frequency of episodes				PLMS	5	4.35%	115
Awareness of occurring catathrenia at home	42	43.75%	96	Bruxism	9	7.83%	115
Awareness occurring every night at home	30	31.25%	96	CSA	3	2.61%	115
Not reported duration	41	32.28%	127	Insomnia	13	11.30%	115
Not reported	31	24.41%	127	Dyspnea	1	0.87%	115
Symptoms				Lack of disorders	22	19.13%	115
Fatigue	34	46.58%	73	Sleeptalking	2	1.74%	115
Morning headaches	43	33.86%	127	Sleepwalking	1	0.87%	115
Low quality of sleep	14	12.17%	115	Narcolepsy with catalepsy	7	6.09%	115
Morning dry mouth	5	4.35%	115	Parasomnias	0	0.00%	114
Excessive daytime sleepiness	9	7.83%	115	Not reported	12	9.45%	127
Symptoms since many years	3	2.61%	115	Vocalization			
Noise	13	11.30%	115	Type I with sinusoidal wave form	17	34.00%	50
Snoring	1	0.87%	115	Type II with semi-rhythmic sawtooth waveform	13	26.00%	50
Awakening/arousal	22	19.13%	115	Lack of atypical sounds	10	20.00%	50
Lack of complaints	2	1.74%	115	Purring	2	4.00%	50
Another pattern of sleep breathing	1	0.87%	115	Animal	1	2.00%	50
Disturbances of bedpartners	1	0.87%	115	Sexual connotation sound	1	2.00%	50
Concerns from family	7	6.09%	115	Not reported	77	60.63%	127
Stridor	1	0.87%	115	Diagnosis			
Breath holding	12	9.45%	127	PSG	122	100.00%	122
Not reported	35	27.56%	127	Polygraphy	2	1.64%	122

*SD*, standard deviation; *NREM*, non-rapid eye movement; *REM*, non-rapid eye movement; *ESS*, Epworth Sleepiness Scale; *PSG*, polysomnography

### Relationship between catathrenia and gender, obesity, health status, and excessive daytime sleepiness

**Fig. 4:**
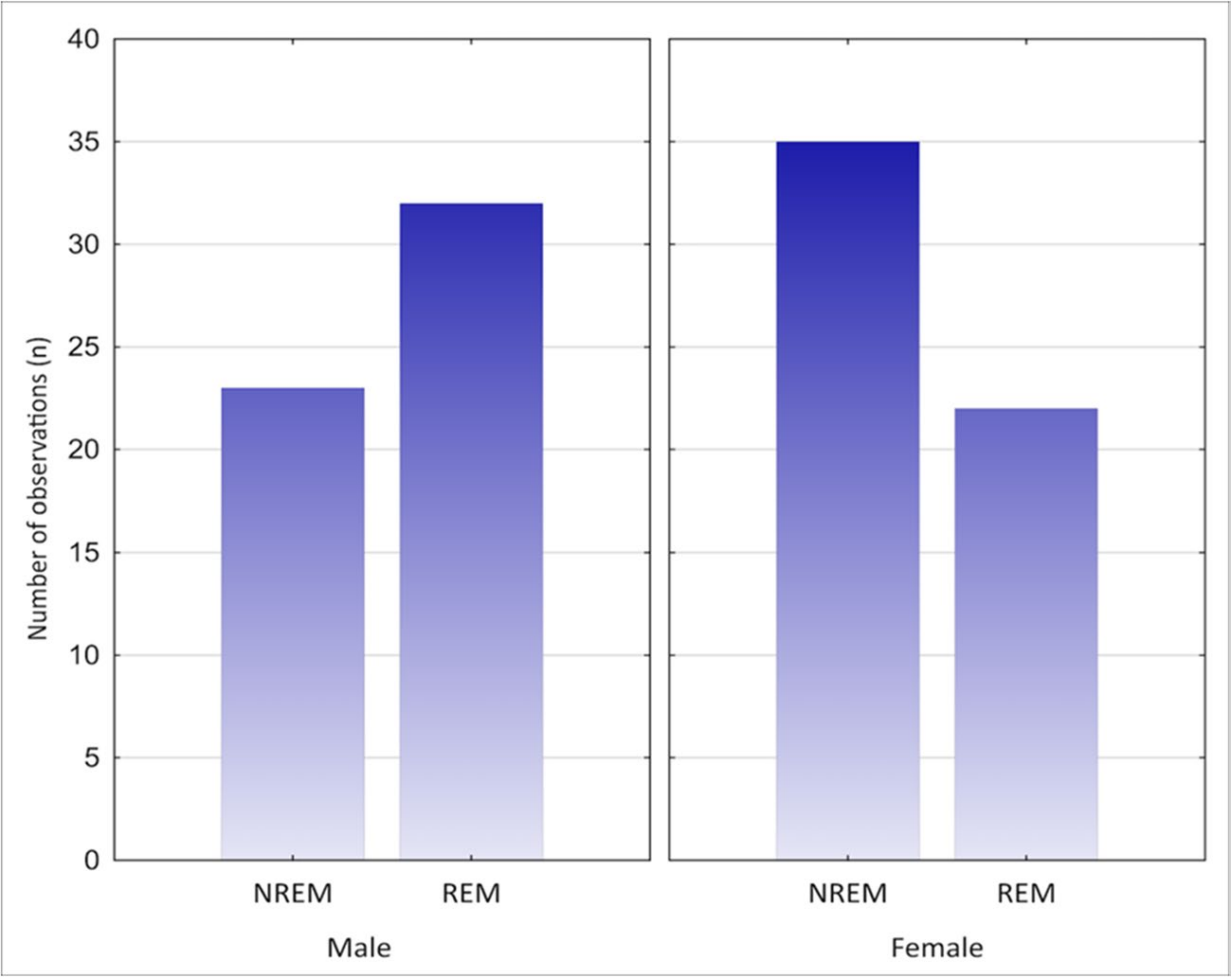
Graph presenting correlation between gender and phase of sleep in catathrenia

We assessed the potential association between gender, health status, obesity, EDS, and particular features of catathrenia such as their predominance in REM vs NREM sleep; minimum and maximum duration of episodes; and ESS score and number of reported occurring episodes at patients’ home per week. Catathrenia occurred in 46% of male and 54% of female participants. Therefore, there is no significant difference in catathrenia prevalence between the genders. However, a significant statistical difference (*p* < 0.05) in episodes of catathrenia between the genders was determined when analyzing their frequency of appearance in different stages of sleep (NREM vs. REM) and their minimal duration. In 60.34% females, catathrenia was more common in the NREM stage of sleep, while 59.26% male the dominant stage of sleep for catathrenia was REM sleep (*n* = 112, chi-square *χ*^2^ = 4.30, df = 1, *p* < 0.05; Fig. 4) The minimal duration of episodes was found to be significantly shorter in the female population (2.19 ± 4.87 s vs 2.25 ± 2.22 s, *p* < 0.05). In obese patients, catathrenia occurred more frequently during the NREM than the REM sleep. This was also statistically significant (*p* < 0.05). On the other hand, patients with BMI normal range (18.5–24.9 kg/m^2^) had episodes occurring more frequently during the REM sleep (*n* = 7 obese vs *n* = 84 non-obese). Obese participants had a significantly shorter duration of the minimal and maximal episodes duration in comparison to non-obese patients (minimal duration of episode: 0.71 ± 0.61 s vs 2.41 ± 4.00 s; maximal duration of episode: 26.51 ± 19.94 s vs 45.14 ± 28.87 s; *p* < 0.05). Comorbid diseases (except sleep disorders) appeared to have the greatest impact on groaning mainly during NREM sleep (*n* = 22 with comorbidities vs. *n* = 22 without them). Patients with comorbidities were also found to have on average, a shorter minimal and maximal duration of each episode (*n* = 29 without comorbidities vs *n* = 11 with them; minimal duration of episode: 2.76 ± 5.48 s vs 2.54 ± 2.50 s; maximal duration of episode: 63.59 ± 29.70 s vs 47.79 ± 21.74 s; *p* < 0.05) and were found to be less sleepy according to the points in the ESS scale, than healthy patients (*n* = 8 without comorbidities vs *n* = 28 with them; 6.07 ± 3.97 s vs 2.21 ± 3.41 s; *p* < 0.05). Additionally, patients who had abnormal ESS (above 10 points, which indicate EDS) were found to have a lower minimal duration of catathrenia episodes (1.44 ± 1.01 s vs 0.73 ± 0.44 s; *p* < 0.05, *n* = 42 ESS below 10 points vs. *n* = 9 ESS above 10 points). These data are shown in Table 3.

**Table 3:** The relationship between particular features of patients and catathrenia episodes

		NREM sleep (%)	REM sleep (%)	Duration—minimum (s)	Duration—maximum (s)	ESS points	Amount of occurring episodes at patients’ home per week
Female	Mean	55.11%	44.22%	2.19	47.08	5.64	5.79
	SD	28.17%	28.69%	4.87	32.62	4.55	1.64
	Number of observations (*n*)	53	53	37	37	58	24
Male	Mean	37.74%	61.22%	2.25	38.65	5.94	6.27
	SD	31.00%	32.14%	2.22	22.92	4.80	1.37
	number of observations (*n*)	38	38	34	34	49	26
*p* value		0.007882	0.012647	0.031153	––––––-	––––-	––––––––-
Obesity	Mean	81.94%	13.00%	0.71	26.51	8.00	5.50
	SD	9.68%	11.91%	0.61	19.94	4.18	2.12
	Number of observations (*n*)	7	7	8	8	9	2
Lack of obesity	Mean	45.02%	54.51%	2.41	45.14	5.57	6.06
	SD	29.88%	30.15%	4.00	28.87	4.66	1.51
	Number of observations (*n*)	84	84	63	63	98	48
*p* value =		0.001606	0.000435	0.008557	0.000497	––––-	–––––––-
Lack of comorbidities	Mean	56.50%	43.32%	2.76	63.59	6.07	7.00
	SD	11.33%	10.74%	5.48	29.70	3.97	0.00
	Number of observations (*n*)	22	22	29	29	28	8
Comorbidities	Mean	64.50%	34.95%	2.54	47.79	2.21	5.83
	SD	28.69%	27.83%	2.50	21.74	3.41	1.83
	Number of observations (*n*)	22	22	11	11	28	6
*p* value =		0.029237	0.029237	0.032045	0.042330	0.000374	–––––––-
ESS—in normal range (below 10 points)	Mean	50.30%	48.90%	1.45	46.43	6.08	5.88
	SD	31.40%	32.36%	1.01	26.74	2.51	1.51
	Number of observations (*n*)	59	59	42	42	66	26
ESS—above 10 points	Mean	37.63%	61.59%	0.73	44.91	13.56	5.67
	SD	28.75%	30.19%	0.55	28.87	2.19	1.87
	Number of observations (*n*)	15	15	9	9	16	9
*p* value =		––––-	––––-	0,019467	––––––-	––––	–––––––

*SD*, standard deviation; *NREM*, non-rapid eye movement; *REM*, non-rapid eye movement; *ESS*, Epworth Sleepiness Scale

### Additional correlations

Using Pearson’s correlation coefficient (*r*), additional associations were identified. Age was inversely correlated with the minimal duration of episodes (*r* =  − 0.34, *p* < 0.05). Symptoms such as abnormal patterns of sleep breathing, as well as the sensation of a dry mouth in the morning, correlated with NREM (*r* = 0.74, *r* = 0.57, *p* < 0.05). Moaning episodes inversely correlated with the REM sleep (*r* =  − 0.30, *p* < 0.05). The maximum duration of catathrenia episodes inversely correlated with the groaning episodes (*r* =  − 0.29) and CPAP therapy (*r* =  − 0.48). The minimal episode duration was also inversely correlated (*r* =  − 0.48, *r* =  − 0.65) with symptoms such as abnormal patterns of sleep breathing and snoring. Sleep bruxism positively correlated with the maximum duration of episodes (*r* = 0.26, *p* < 0.05). Insomnia also positively correlated with symptom groaning (*r* = 0.49, *p* < 0.05). Frequency of episodes at patients’ home, including those episodes reported every night at home, was positively correlated with the duration of REM sleep (*r* = 0.61, *r* = 0.38, *p* < 0.05). During CPAP therapy, catathrenia episodes correlated with the duration of NREM sleep (*r* = 0.69, *p* < 0.05).

### Risk of bias

The majority of the reviewed studies were case reports. Unfortunately, 9 of the 14 case reports were determined to have a high risk of bias [17, 27, 28, 30, 32, 46, 50, 51]. Only one study in this group [49] was determined to have a low risk of bias. The remaining case reports [18, 33, 53, 54] were determined to have a moderate risk of bias. Similarly, the majority of the case series publications (6 out of 10) was deemed to have a high risk of bias [22, 24, 37, 40, 45, 47]. The remaining 4 papers had an overall moderate risk of bias [19, 25, 38, 41]. Unfortunately, not a single case series was deemed to have a low risk of bias. Using the ROBINS-I tool, 6 cohorts [16, 26, 31, 42–44] and 1 case–control study [39] were inspected. One study was found to have a serious risk of bias [16]. And only 1 study [43] was determined to have a low risk of bias according the ROBIN-I criteria. The rest of the studies (5 out of 7) were determined to have a moderate risk of bias [26, 31, 39, 42, 44]. Detailed descriptions for the risk of bias assessment have been presented in Table 4 for case reports, Table 5 for case series, and Table 6 for observational (cohort and case–control) studies.

**Table 4:** Assessment of risk of bias for case reports according to the Joanna Briggs Institute (JBI) checklist

Authors	Q1	Q2	Q3	Q4	Q5	Q6	Q7	Q8	Overall assessment
Villafuerte-Trisolini B et al. [27]	No	Yes	No	Yes	No	No	No	Yes	High
Steinig J et al. [46]	No	Yes	No	Yes	Yes	No	Yes	No	High
Songu M et al. [33]	No	Yes	Yes	Yes	Yes	Yes	No	Yes	Moderate
Ramar K et al. [30]	No	Yes	No	Yes	Yes	No	No	Yes	High
Argollo NS et al. [18]	No	Yes	Yes	Yes	Yes	Yes	No	Yes	Moderate
Manconi M et al. [49]	Yes	Yes	Yes	Yes	Yes	Yes	No	Yes	Low
Grigg-Damberger M et al. [50]	No	Yes	Yes	Yes	No	No	No	Yes	High
Romigi A et al. [32]	No	Yes	Yes	Yes	No	No	No	No	High
Bansal R et al. [51]	Yes	Yes	Yes	Yes	No	No	No	Yes	High
Motojima T et al. [52]	No	Yes	No	Yes	No	No	No	Yes	High
Gómez T et al. [53]	No	Yes	Yes	Yes	Yes	Yes	No	Yes	Moderate
Siddiqui F et al. [17]	No	Yes	Yes	Yes	Yes	No	No	No	High
Bar C et al. [28]	No	Yes	Yes	Yes	Yes	No	No	Yes	High
Carbajal-Mamani S et al. [54]	No	Yes	Yes	Yes	Yes	Yes	No	Yes	Moderate

Q1. Were patient’s demographic characteristics clearly described?

Q2. Was the patient’s history clearly described and presented as a timeline?

Q3. Was the current clinical condition of the patient on presentation clearly described?

Q4. Were diagnostic tests or methods and the results clearly described?

Q5. Was the intervention(s) or treatment procedure(s) clearly described?

Q6. Was the post-intervention clinical condition clearly described?

Q7. Were adverse events (harms) or unanticipated events identified and described?

Q8. Does the case report provide takeaway lessons?

**Table 5:** Assessment of risk of bias for case series according to the Joanna Briggs Institute (JBI) checklist

Authors	Q1	Q2	Q3	Q4	Q5	Q6	Q7	Q8	Q9	Q10	Overall assessment
Prihodova I et al. [37]	No	Yes	Yes	No	No	No	No	Yes	No	Yes	High
Yu M et al. [25]	Yes	Yes	Yes	Yes	Yes	No	Yes	No	No	Yes	Moderate
Oldani A et al. [38]	No	Yes	Yes	Yes	Yes	No	Yes	Yes	No	Yes	Moderate
Tereshko Y et al. [47]	No	Yes	Yes	No	No	No	Yes	Yes	No	No	High
Yu M et al. [19]	Yes	Yes	Yes	Yes	Yes	Yes	Yes	No	No	Yes	Moderate
Guilleminault C et al. [40]	Yes	Yes	Yes	No	No	Yes	Yes	No	No	No	High
Koo DL et al. [41]	No	Yes	Yes	Yes	Yes	Yes	Yes	No	No	Yes	Moderate
Vetrugno R et al. [22]	No	No	Yes	No	No	No	Yes	No	No	No	High
Kazaglis L et al. [45]	No	Yes	Yes	No	No	Yes	Yes	No	No	No	High
Iriarte J et al. [48]	No	Yes	Yes	No	No	No	Yes	No	No	Yes	High

Q1. Were there clear criteria for inclusion in the case series?

Q2. Was the condition measured in a standard, reliable way for all participants included in the case series?

Q3. Were valid methods used for identification of the condition for all participants included in the case series?

Q4. Did the case series have consecutive inclusion of participants?

Q5. Did the case series have complete inclusion of participants?

Q6. Was there clear reporting of the demographics of the participants in the study?

Q7. Was there clear reporting of clinical information of the participants?

Q8. Were the outcomes or follow-up results of cases clearly reported?

Q9. Was there clear reporting of the presenting site(s)/clinic(s) demographic information?

Q10. Was statistical analysis appropriate?

**Table 6:** Assessment of risk of bias for observational studies according to the ROBINS-I domains

Authors	D1	D2	D3	D4	D5	D6	D7	Overall assessment
Abbasi AA et al. [31]	Low	Low	Low	Low	Moderate	Moderate	Low	Moderate
Alonso J et al. [16]	Moderate	Serious	Moderate	Moderate	Moderate	Moderate	Low	Serious
Overland B et al. [42]	Low	Low	Low	Moderate	Low	Moderate	Low	Moderate
Drakatos P et al. [43]	Low	Low	Low	Low	Moderate	Low	Low	Low
Poli F et al. [26]	Moderate	Low	Low	Moderate	Moderate	Low	Low	Moderate
Pérez-Carbonell L et al. [44]	Low	Moderate	Low	Moderate	Low	Low	Low	Moderate
Vetrugno R et al. [39]	Moderate	Low	Low	Low	Low	Moderate	Moderate	Moderate

D1. Bias due to confounding

D2. Bias in selection of participants into the study

D3. Bias in classification of interventions

D4. Bias due to deviations from intended interventions

D5. Bias due to missing data

D6. Bias in measurement of outcomes

D7. Bias in selection of the reported result

## Discussion

Our systematic review set out to organize existing data on catathrenia, to help us better understand this disorder. The primary objective was to identify the most common features of catathrenia. The second objective was to determine the potential association between catathrenia and gender, symptoms, and particular sleep stages. The majority of the included studies was made up of case reports and case series. Nevertheless, some conclusions can be drawn.

First of all, catathrenia was reported in a wide range of age, e.g., in the pediatric group of patients (in 4, 10, or 12 years old) [23, 28, 50, 52] or in elderly participants (including in 76-year-old patients), or mean age range of 47.9 ± 15.1 years [24, 31, 44]. The mean age of patients included in this systemic review was determined to be 31.45 ± 14.66 years. However, it is important to note that this is the calculated age at which the diagnostic process was conducted. It does not describe the mean age of atypical symptom onset, which could appear many years before the diagnosis was finally made [19, 50]. According to the ICSD-2 classification, the onset of nocturnal moaning was estimated to appear in childhood on average 9 ± 10 years [24]. Increasing age was found to be associated with a reduction in the minimum duration of these episodes; however, this was determined to be a weak correlation. Aging is often described as a risk factor for diseases such as cardiovascular disease or obesity [55, 56]; thus, we would expect that age would lengthen the duration of the episodes or in the very least have an additional negative influence on features of catathrenia. However, age seems to be a mitigating factor in this condition. It is also possible that with higher age sleep is easily disrupted by episodes of catathrenia which results in awakenings or arousals shortening the duration of episodes.

Previously, it was estimated that episodes of catathrenia would last around 2–49 s; however, our literature review uncovered that there have been episodes recorded which have either a much longer duration [32] or shorter than expected duration [28]. Our review determined that the minimal duration can be estimated to be around 2.22 ± 3.81 s, while the maximal duration was recorded to be around 43.04 ± 28.51 s, which is close to the range indicated in ICSD-2 [57].

As previously there was reported [16] that gender does not influence catathrenia and our analysis confirmed this. When considering the prevalence of groaning in particular phases of sleep, some of the reviewed studies indicated that catathrenia appeared predominantly during the REM sleep [37–40, 42]. It is worth mentioning that catathrenia was originally classified as REM parasomnia [57]. However, in literature, we could find data describing groaning episodes during the NREM sleep, while other articles even reported episodes only during NREM sleep [19, 26, 31]. Therefore, Abbasi et al. [31] divided the nocturnal episodes into two main subtypes, e.g., typical moaning, which met the ICSD-2 criteria of these episodes appearing during REM vs. atypical groaning, which dominated the NREM phase of sleep. In fact, our review found that episodes of catathrenia appeared more frequently during the NREM rather than during the REM sleep (49.57% of episodes in NREM sleep vs 46% of episodes in REM sleep). Furthermore, the incidence of catathrenia episodes in REM or in NREM sleep was different among genders. Catathrenia appeared more frequently during the NREM stage of sleep in females, while it was found to dominate the REM sleep in male participants. These relationships have thus far never been mentioned in literature. However, we have to question, whether these differences are clinically significant. If we take into account that females were determined to have a shorter minimal duration of episodes than men, we may consider catathrenia as a sex-specific condition. Overall, the prevalence of sleep disorders is greater in the female, than in the male population [58, 59], although this prevalence cannot be deemed to be significant. Nevertheless, the clinical picture suggests that females have more correlations with features of groaning. These variations however require further research.

Until recently, catathrenia was considered to be a condition with several potential mechanisms; however, it has yet to be linked to specific diseases. It was also believed to have only caused significant symptoms [16, 25]. Nocturnal groaning has sometimes been associated with sleep bruxism (SB) or OSA [25, 33, 49]. Hao et al. [60] however did find that groaning patients had completely different anatomical characteristics compared to patients with OSA, e.g., these patients were found to have a wide upper airway and large skeleton. We only found a weak relationship between SB and a prolonged maximal episode duration. Interestingly, among sleep disorders, neither OSA nor SB occurred concomitantly with catathrenia as frequently as insomnia did in our review. More intensified groaning episodes were observed in patients experienced insomnia, although this correlation was deemed to be moderate and difficult to explain. Insomnia reduces the amount of sleep and catathrenia occurs during sleep without awareness. Insomnia shorts overall periods of sleep, thus comorbid catathrenia had to have intensified episodes to would have observed catathrenia and this relationship in patients. However, the majority of patients with catathrenia did not have any other sleep problems. In comparison to OSA, Buyse et al. [61] recently discovered that catathrenia episode before severe OSA episode may slow the oxygen desaturation drop and additionally, catathrenia arousals were connected with “rescue” breathing after OSA episode. Usually in typic catathrenia, arousals after the groaning incidence were observed independently [43]. It seems that catathrenia does not change the sleep structure in contrast to OSA [62]. However, during PSG, catathrenia may imitate the central sleep apnea [16, 17] and this may be the cause of rare reporting catathrenia. There is a need to conduct further studies about the exact relationship between catathrenia and sleep apnea.

Aside from sleep disorders, we also focused on the association between catathrenia and systemic diseases; however, no such relationship was uncovered. It is worth emphasizing though that only 46.58% of participants were without comorbidities and the rest of them had comorbidities. In literature, catathrenia has been described as a condition in serious diseases such as fatal insomnia [44], Pitt–Hopkins syndrome [52], or mental retardation [37]. In our analysis, we determined that the majority of the comorbidities was made up of general disorders, such as allergic rhinitis, depression, attention-deficit hyperactivity disorder (ADHD), or obesity [16, 37, 45]. Our review also determined that comorbidities (excluding sleep disorders) played a role in the presentation of groaning during the NREM sleep; they shortened the minimal and maximal episode duration and scored less points in ESS. However, we were unable to specify which comorbidities influenced these results. Obesity was found to be a predisposing factor in the appearance of moaning during the NREM sleep and resulted in shorter episode durations. Similar to aging, obesity is also responsible for health deterioration and leads to chronic diseases and increased mortality [63]. Interestingly, though obesity was found to reduce the minimal episode duration. We do not know why the above conditions had an inverse effect on certain features of catathrenia, in contrast to other disorders. These results have not been presented in literature; therefore, to determine the association between catathrenia and health status, further studies have to be conducted.

Our study also investigated numerous symptoms reported in patients diagnosed with catathrenia. The most frequently reported complaints included awareness of disturbing to bed partners, snoring, groaning, atypical nocturnal sounds, and EDS. However, according to the ESS, which is considered to be a reliable scale used in the diagnosis of excessive sleepiness [64], albeit subjective, there were 5.78 ± 4.65 points in ESS. However, in order to diagnose pathological sleepiness during the day, patients are required to obtain over 10 points in the ESS [65]. We discovered that participants who got over 10 points in the ESS had a lower minimal duration of moaning episodes. Patients also frequently reported family concerns over their health, the sensation of a dry mouth in the mornings, or nocturnal noises. Snoring and symptoms such as abnormal patterns of sleep breathing were found to prolong the minimal time of the catathrenia episodes. One of the most reported complaints of catathrenia embraced sensation of a dry mouth and EDS which may be also an OSA symptom [66–69]. However, according to our analysis, OSA is not the most frequent among sleep disorders co-existing with catathrenia. Therefore, it is an interesting issue whether there exists a common pathway for OSA and catathrenia manifested by similar symptoms. Unfortunately, literature did not describe potential common mechanisms for OSA and catathrenia. But Songu et al. showed CPAP treatment improved catathrenia [33]; thus, a common pathway may exist. Moreover, similar symptoms of catathrenia without reporting nocturnal groaning may imitate the OSA disorder and without PSG conducting, catathrenia may be misdiagnosed. Nocturnal behaviors were reported by patients’ families, because patients are often unaware of the moaning [24]. Therefore, the reported complaints have to be taken into consideration very carefully, especially in conditions such as catathrenia, which we do not, as yet, fully understand. Families could also embellish the reported symptoms. One such family had reported hearing groaning from their son, that was so loud, they were afraid to go camping due to concerns that they may be attacked by bears, who might think that the sound is coming from a hurt, dying animal [50]. Other descriptions of groaning included purring or sexual connotations [28, 40]. According to an objective analysis of episode vocalization, the most common vocalization was the type I sound with a sinusoidal waveform, followed by the type II sound with a semi-rhythmic sawtooth waveform, which consistently indicated the vocal origin of the episodes [19]. The lack of atypical sounds was confirmed during PSG study.

Almost half the analyzed population reported their awareness about occurring catathrenia episodes at home. In fact, according to patients’ history, 30% of the participants experienced episodes almost every single night at home. These episodes were determined to have a moderate relationship with the REM sleep, which is partially confirmed that occurring catathrenia episodes according to ICSD-3. This is in contrast to our overall results, where episodes of catathrenia appeared more frequently during the NREM cycle. To diagnose catathrenia, PSG was used in the majority of the studied patients. Numerous treatment modalities were described including CPAP therapy, pharmacotherapy, the utilization of a mandibular advancement device (MAD), and botulinum toxin. CPAP therapy was the most commonly used form of treatment and is the standard therapy for sleep-breathing disorders (SDB), allowing to improve patient quality of life, reducing EDS and causing a reduction in blood pressure values [70]. Patients with catathrenia who underwent CPAP therapy were found to have episodes that occurred most frequently during the NREM sleep phase. Our analysis found that CPAP therapy also caused a reduction in the maximal duration of the groaning episodes, which may indicate the respiratory origin of catathrenia.

Despite conducting a reliable analysis of the available data regarding catathrenia, our study does have a number of limitations. First of all, catathrenia is a rare condition and our analysis was based on few papers, which did not always contain the entire patient’s history and did not thoroughly analyze the features of catathrenia. Almost every study focused on different features of catathrenia. Therefore, each parameter that was analyzed had a different number of participants. The largest amount of data came from single studies. However, even if the studies presented a cohort of patients, these publications were deemed to have a high or moderate risk of bias. There were also a number of papers that embraced the subject of catathrenia; however, we could not classify these studies even as case reports. As a result, we could not extract their data and use it in our analysis. Unfortunately, due to a lack of access to some new studies focusing on catathrenia, our article does not contain some important pieces of knowledge. Taking into consideration all of these limitations, future studies will have to include the detailed features of catathrenia in cohort patients and explain the potential long-term consequences of catathrenia on patients’ health. These studies will also have to take into consideration the relationship between patients’ status and catathrenia features.

## Conclusions

Catathrenia occurs with similar frequency in both genders. The most frequent complaints of the patients with catathrenia are groaning or other atypical nocturnal sounds, awareness of disturbing bedpartners, snoring, and daytime somnolence—although not confirmed by ESS. The episodes of catathrenia occur more frequently in NREM than in REM sleep. However, at home episodes occurred according to ICSD-3 in REM sleep. Obesity and aging reduce the duration of these episodes. Catathrenia may be considered as a sex-specific condition, because as compared with males in females its episodes occur more frequently in NREM than in REM sleep and are characterized by longer minimal duration. There is no influence of the comorbidities on the incidence of catathrenia. However, some systemic diseases may be associated with shorter minimal and maximal duration of its episodes, as well as with lower ESS score. The effects of CPAP treatment leading to shortening of the maximal duration of the episodes of catathrenia may indicate the respiratory origin of catathrenia.

### Supplementary Information

Below is the link to the electronic supplementary material.

Supplementary file1 (DOCX 49 KB)

## Data Availability

Data available on request.

## Abbreviations

ICSD-2: International Classification of Sleep Disorders, version 2
ICSD-3: International Classification of Sleep Disorders, version 3
REM: Rapid eye movement
NREM: Non-rapid eye movement
PSG: Polysomnography
OSA: Obstructive sleep apnea
PRISMA 2020: Preferred Reporting Items for Systematic Reviews and Meta-Analyses 2020
ESS: Epworth Sleepiness Scale
JBI: The Joanna Briggs Institute
SD: Standard deviation
EDS: Excessive daytime sleepiness
BMI: Body mass index
CPAP: Continuous Positive Airway Pressure
SB: Sleep bruxism
ADHD: Attention-deficit hyperactivity disorder
Fig: Figure
MAD: Mandibular advancement device
