# Correction to: The clinical characteristic of catathrenia: a new look at an old issue—a systematic review of existing literature

**DOI:** 10.1007/s11325-024-03088-z

**Published:** 2024-06-27

**Authors:** Bartlomiej Blaszczyk, Adam Wichniak, Mieszko Wieckiewicz, Anna Brzecka, Dorian Nowacki, Monika Michalek-Zrabkowska, Gabriella Lachowicz, Grzegorz Mazur, Helena Martynowicz

**Affiliations:** 1 Student Research Club No K133, Faculty of Medicine, Wroclaw Medical University, 50-556, Wroclaw, Poland; 2 Third Department of Psychiatry and Sleep Medicine Centre, Institute of Psychiatry and Neurology, Warsaw, 02-957 Poland; 3 Department of Experimental Dentistry, Wroclaw Medical University, 50-425, Wroclaw, Poland; 4 Department of Pulmonology and Lung Cancer, Wroclaw Medical University, 53-439, Wroclaw, Poland; 5 Department of Human Nutrition, Wroclaw University of Environmental and Life Sciences, Wroclaw, 51-630 Poland; 6 Department of Internal Medicine, Occupational Diseases, Hypertension and Clinical Oncology, Wroclaw Medical University, Wroclaw, 50-556 Poland

**Correction to: Sleep and Breathing**

10.1007/s11325-024-03033-0

In the originally published version of this article, the name of the third author was incorrectly spelled as “Mieszko Wieckiewcz”. The correct name is “Mieszko Wieckiewicz”.

Also, the affiliation for authors Monika Michalek-Zrabkowska, Gabriella Lachowicz, Grzegorz Mazur, and Helena Martynowicz was incorrectly given as “Student Research Club No K133, Faculty of Medicine, Wroclaw Medical University, 50-556, Wroclaw, Poland”. The correct affiliation of these authors is “Department of Internal Medicine, Occupational Diseases, Hypertension and Clinical Oncology, Wroclaw Medical University, 50-556 Wroclaw, Poland.”

The authors regret for these mistakes.

The original article has been corrected.

