# Supplementary material for: The clinical characteristic of catathrenia: a new look at an old issue—a systematic review of existing literature
Source: Sleep Breath. 2024 May 17;28(4):1523–37. doi: 10.1007/s11325-024-03033-0 (PMC11303500; doi:10.1007/s11325-024-03033-0)
Supplement: Supplementary file 1 — Supplementary file1 (DOCX 49 KB) [file 11325_2024_3033_MOESM1_ESM.docx]

Table 1. The detailed characteristics of patients included in studies.

| Study authors | Patients number and gender | Body mass index (BMI) | Age | Comorbid diseases | Sympthoms | Excessive daytime sleepiness (EDS) | Methods used to diagnose catathrenia | Concomitant sleep disorders | | Sleep stage during moaning | Frequency of groaning | | | Duration | Vocal sound | Treatment |
| --- | --- | --- | --- | --- | --- | --- | --- | --- | --- | --- | --- | --- | --- | --- | --- | --- |
| Abbasi AA et al. [31] | 5 females, 5 males, | 29.4±  8.8 kg/m2 | 46.2±22.5 years old | Not reported | 9 times snoring and sleep-disordered breathing, 3 times  groaning and moaning | 9.8 ± 4.3 points in ESS | 10 times PSG | 3 times OSA | | NREM – 85.6 %, REM – 8.5%, awaking – 6.1% | Not reported | | | 0.4 - 21.4 s, 86 were below 2s | Not reported | 4 times CPAP |
| Alonso J et al. [16] | 27 females, 20 males | 24.8 ± 4.6 kg/m2 | 40.2 ± 10.9 years old | 21 anxiety or depression, 3 gastroesophageal reflux,  2 hypothyroidism, 2 asthma,  2 psychiatric disorder, 1 high blood pressure, 1 migraine, 1 diabetes | 39 unrefreshing sleep, 36 fatigue, 33 decreased  daytime alertness, 29 decreased concentration or  memory, 24 witnessed gasping or choking episodes, 25 waking up at night to urinate  21 insomnia sudden waking with breath  holding; 15 gasping, or choking, | 8.3 ± 5.0 points in ESS | 29 times PSG,  2 ambulatory  sleep studies, 3 times without examinations, 12 times self diagnosis based on sympthoms | 10 times OSA, 27 times parasomnias | | REM in 2 cases, the rest are not reported | 6.56 ± 1.15 episodes per week at home | | | Not reported | Not reported | 11 times CPAP |
| Prihodova I et al. [37] | 3 females, 5 males | Not reported | 23 ± 7.1 years old | 2 mild mental retardation, 2 ADHD, 2 depression, 1 allergic rhinitis, 1 craniocerebral trauma, 1 focal epilepsy | 4 excessive daytime sleepiness, 5 fatigue, 4 unrefreshing sleep, 1 difficult morning awakening, 1 irritable mood | Not reported | 8 times PSG | 4 times bruxism, 4 ronchopathy, times insomnia, 2 nocturnal dyspnea, 1 sleepwalking, 1 nocturnal  awakenings | | REM -76.5%, NREM – 21.5%, | Not reported | | | 2–46 s mean duration 12.5 s | Not reported | Pharmacoteraphy |
| Yu M et al. [19] | 15 females, 8 males | 22.3 ± 5.1 kg/m2 | 29.6 ± 10.0 years old | 1 bilateral tonsillectomy and pharyngoplasty,  1 Ménière’s disease, 2 snoring | 15 disturbances of bedpartners or roommates, 10 concerns from family members. dry mouth in the morning, unrefreshing sleep, a lack of concentration  during the day | 4 times ESS > 10 points, mean 8.3 ± 2.3 points in ESS | 23 times PSG | 5 times OSA, 4 times bruxism | | REM – 40%, NREM – 60% | 50 (32, 131) episodes during PSG | | | 1.3-74.9 s, mean duration 11.4 ± 4.6s, | 13 sounds type I with sinusoidal wave form, 10 sounds  type II with semi-rhythmic sawtooth waveform | Not reported |
| Oldani A et al. [38] | 8 females, 13 males | Not reported | 31.4 ±  8.1 years old | Not reported | Not reported | 5 times ESS > 10 points | 16 times PSG | 2 times bruxism | | REM – 89%,  NREM – 11% | 3.0±1.8 episodes during PSG, 5.9 ± 1.5 episodes per week at home | | | Not reported | Not reported | 2 clonazepam, 1 gabapentin  1 pramipexole, 1 trazodone |
| Yu M et al. [25] | 18 females, 12 males | 22.0 ± 2.7 kg/m2 | average 31.0 years old | Not reported | 30 groaning  noises during sleep, 11 unrefreshing sleep, 7 daytime  fatigue, 6 decreased memory and concentration | 14 times ESS > 10 points | 30 times PSG | 6 times OSA | | REM – 36.7%, NREM – 50%, REM and NREM – 13.3% | Range 0.53–57.6 episodes per hour during PSG | | | Not reported | Not reported | Mandibular advancement  Device (MAD) therapy |
| Vetrugno R et al. [39] | 5 females, 5 males | 22.2 ± 1.7 kg/m2 | 27 ± 7.4 years old | Not reported | Not reported | 3 ± 2 points in ESS | 10 times PSG | Not reported | | NREM 55.5 ± 5.1%, NREM 22.3 ± 3.8%, REM 23.1 ± 5.6% | Almost every night at home | | | 2–20 s | Lack of atypical sounds | Not reported |
| Guilleminault C et al. [40] | 7 females | Average 23 kg/m2 | Mean 26.7 years old | 3 wisdom teeth  extracted, 3 orthodontics, 2 nasal allergies, 1 depression, 1 bruxism | 5 dry  mouth, 6 mouth breathing, 1 snoring, 1 fatigue, 1 morning headache, 1 bouts of insomnia | 4.4 ± 0.98 points in ESS | 7 times PSG | Lack of concomitant sleep disorders | | Not reported | Not reported | | | Not reported | 1 sound emitted was quite loud and produced a distinct sexual connotation | 7 times CPAP |
| Koo DL et al. [41] | 5 females | Mean 20.5 kg/m2 | Mean 31.0 years old | Not reported | Not reported | 9.2 ± 4.6 points in ESS | 5 times PSG | Lack of concomitant sleep disorders | | REM - 61.9%, NREM - 38.1% | Mean 3.6 episodes per week at home | | | 0.3–15.1 s, mean duration on 5.6 s | 3 sounds type I with sinusoidal wave form, 2 sounds  type II with semi-rhythmic sawtooth waveform | Not reported |
| Overland B et al. [42] | 4 males | 26 ± 2.5 kg/m2 | 34.5 ± 4 years old | Not reported | Not reported | 7.3 ± 2.3 points in ESS | 4 times PSG | 1 times OSA, 1 times PLMS | | REM – 100% | 1.7 -17.1 episodes per hours during PSG | | | 4 -38 s | Not reported | 1 CPAP |
| Drakatos P et al. [43] | 15 female, 23 males | 25.9 ± 5.3 kg/m2 | 33.1 ± 7.7 years old | Not reported | 20 moaning, 19 breath holding  7 snoring,  17 sleepiness,  2 humming, 3 unrefreshing sleep, 2 grunting, 2 sleepwalking | 10.1 ± 5.3 points in ESS | 38 times PSG | 12 times OSA, | | REM – 91%, NREM – 19% | 11.4±10.5 episodes during PSG | | | 53.2±46.5 s | Not nreported | 9 CPAP, 6 MAD,  1 Provent,  5 Clonazepam,  5 Zopiclone,  6 cognitive behavioral therapy for insomnia,  9 No treatment |
| Poli F et al. [26] | 5 females, 2 males | 21.9 ± 3.5 kg/m2 | 25.7 ± 14.6 years old | 7 Narcolepsy with catalepsy | Noisy breathing during sleep  that differed from snoring or a strange sound while breathing during sleep | Not reported | 7 times PSG | 7 times narcolepsy with catalepsy | | REM – 20%, NREM – 80% | Not reported | | | Not reported | Not reported | Not reported |
| Pérez-Carbonell L et al. [44] | 6 females, 2 males | Not reported | 51.4 ± 15.2 years old | 8 Fatal insomnia, 7 cognitive imparement, 4 gait disturbance, 3 dysphagia, 4 diplopia, 3 dysphonia, 2 parkinsonism, 3 dysmetria, 4 perspiration, | Not reported | Not reported | 8 times PSG | 6 insomnia, 3 excessive daytime sleepiness, 3 CSA, 4 PLMS, | | Not reported | 3 times > 20 episodes during PSG, 5 time > 4 episodes during PSG | | | Not reported | 7 stridor, 7 expiratory groaning, 6 hiccups, 2 rhythmic expiratory vocalizations | Not reported |
| Vetrugno R et al. [22] | 1 female, 3 males | Normal | 15 years old | Healthy | Moans and groans since age 8 | Not reported | 4 times PSG | Absent | | REM – mainly 100%, NREM – 1 episode | Almost every night at home | | | 2 – 20 s | Mournful sounds and groans | Not reported |
|  |  |  | 23 years old |  | Groans since age 16 |  |  |  |  |  |  |  |  |  |  |  |
|  |  |  | 25 years old |  | Nocturnal groaning since age 5 |  |  |  |  |  |  |  |  |  |  |  |
|  |  |  | 25 years old |  | Nocturnal groaning since age 14 |  |  |  |  |  |  |  |  |  |  |  |
| Kazaglis L et al. [45] | 1 male | 26.8 kg/m2 | 52 years old | Healthy | Snoring and episodes of nocturnal breathholding | 3 points in ESS | Home sleep apnea test (HSAT), PSG | OSA | | NREM – 100% | Not reported | | | Not reported | Not reported | Not reported |
|  | 1 female | 34.3 kg/m2 | 18 years old | Obesity | Nocturnal moaning episodes | 6 points in ESS |  | Absent | | REM – 100% |  |  |  |  |  |  |
| Villafuerte-Trisolini B et al. [27] | 1 male | Not reported | 10 years old | Attention  deficit hyperactivity disorder (ADHD) | Lack of sleep complaints | Not reported | PSG | Cyclic alternating pattern (CAP), bruxism | NREM – 100% | | | Almost every night at home | Not reported | | Vocal groaning  sound like purring | CPAP |
| Steinig J et al. [46] | 1 male | 25 kg/m2 | 33 years old | Not reported | Non-restorative sleep and occasional  increased daytime tiredness | 12 points in ESS | PSG | Absent | REM only | | | Not reported | Maximum 32 s | | prolonged high-pitched sound – whining and squealing | CPAP |
| Songu M et al. [33] | 1 female | 36 kg/m2 | 40 years old | Cardiology problems | Restless sleep and tiredness during  the daytime | Not reported | PSG | OSA | REM, NREM, | | | Several time in every night | 2–10 s | | Not reported | CPAP |
| Ramar K et al. [30] | 1 male | 25 kg/m2 | 41 years old | Healthy | Abnormal breath sounds | 6 points in ESS | PSG | Absent | REM – 100% | | | Almost every night at home | Not reported | | funny sounds | Not reported |
| Tereshko Y et al. [47] | 1 female | 18.7 kg/m2 | 31 years old | Not reported | Frequent nocturnal awakenings and moanings during sleep | Not reported | PSG | Absent | REM – mainly, NREM - occasional | | 5.07 episodes per hour during PSG | | Not reported | | Not reported | Intramuscular injection of botulinum toxin type A (BoNT/A |
|  | 1 male | 27 kg/m2 | 28 years old | Insomnia secondary to anxiety | Loud moanings during sleep |  |  | Absent | REM – 100% | | 2.13 episodes per hour during PSG | |  |  |  |  |
| Iriarte J et al. [48] | 1 female | Not reported | 62 years old | Not reported | Uncomfortable noise for her  family and the neighbours,  sporadic dry mouth | Not reported | PSG | Not reorted | Not reorted | | Not reorted | | 8–15 s | | 1 sounds type I with sinusoidal wave form | Not reported |
|  | 1 male | Not reported | 65 years old | Healthy | Production of a noise for  the last 30 years at least |  |  |  |  |  |  |  | Not reported | | 1 sounds  type mixed I–II with both waveform |  |
| Argollo NS et al [18] | 1 male | Not reported | 6 years old | Allergic rhinitis | Snoring and night agitation | Not reported | PSG | OSA | NREM – 100% | | 4 episodes during PSG | | 10 - 15s | | Not reported | Not reported |
| Manconi M et al. [49] | 1 male | Not reported | 31 years old | Healthy | Nocturnal vocal sound, orofacial discomfort, such as  pain, fatigue, muscular tension | Not reported | PSG | Bruxism | NREM – 100% | | Almost every night at home, 5.5 episodes per hour during PSG | | Not reported | | Not reported | CPAP, clonazepam |
| Grigg-Damberger M et al. [50] | 1 male | 18.8 kg/m2 | 12 years old | Asthma, rethrognathia, congenital talipes equinovarus | Since 9 years the patient has exhibited periods of groaning and moaning | Not reported | PSG | OSA | NREM – 100% | | 1 hour groaning almost every night, | | Not reported | | Moans so loudly that parents are afraid bears will attack us when we’re camping, thinking he’s ahurt, dying animal | CPAP |
| Romigi A et al. [32] | 1 female | Not reported | 15 years old | Healthy | Episodes of nocturnal  unusual sounds occurring in clusters | Not reported | Polygrahy, PSG | Absent | NREM, REM | | Almost every night at home, | | 30 - 154 s | | Not reported | Not reported |
| Bansal R et al. [51] | 1 female | 30.3 kg/m2 | 54 years old | Borderline hypertension | Abnormal sounds during sleep since the age of 17, morning dry mouth, occasional morning headaches,  excessive daytime sleepiness | Not reported | PSG | OSA | REM | | 3 or 4  nights per week at home ; episodes  occur with greater frequency during stress | | Not reported | | Consistent  tone and volume occur sporadically but  in clusters | CPAP |
| Motojima T et al. [52] | 1 female | Not reported | 4 years old | Pitt–Hopkins syndrome | Loud groaning  sounds during episodes of prolonged expiration, and tended to  be exacerbated in winter and improved in summer since 3 years | Not reported | PSG | Insomnia | NREM, REM | | Every night from midnight to early morning at home | | Not reported | | Not reported | Not reported |
| Gómez T et al. [53] | 1 female | 24 kg/m2 | 22 years old | Healthy | Nocturnal groaning and arousals,  low quality of  sleep, daytime tiredness, and morning headache | Not reported | PSG, polygraphy | Absent | REM mostly | | 18 episodes during whole PSG | | Not reported | | Not reported | CPAP |
| Siddiqui F et al. [17] | 1 male | Not reported | 13 years old | Healthy | Recurrent episodes of witnessed apneas, drowsy and tired during the daytime | Not reported | PSG | Absent | NREM – 100% | | Not reported | | 10 – 12 s | | Not reported | Not reported |
| Bar C et al. [28] | 1 male | Not reported | 4 years old | Healthy | Recurrent episodes of  unusual nocturnal sounds | Not reorted | PSG | Absent | NREM – 100% | | Almost every night at home, 3 episodes during PSG | | 2–8 s | | Purring  sound | Not reported |
| Carbajal-Mamani S et al. [54] | 1 female | Normal | 25 years old | Anxiety, depression, excessive  sleepiness | Episodes of  moaning and knuckle cracking during sleep | Not reported | PSG | Absent | NREM – 100% | | At least 3 nights per week at home, 1 episode during PSG | | Not reported | | Not reported | Not reported |
